# Admixture mapping in two Mexican samples identifies significant associations of locus ancestry with triglyceride levels in the *BUD13/ZNF259/APOA5* region and fine mapping points to rs964184 as the main driver of the association signal

**DOI:** 10.1371/journal.pone.0172880

**Published:** 2017-02-28

**Authors:** Esteban J. Parra, Andrew Mazurek, Christopher R. Gignoux, Alexandra Sockell, Michael Agostino, Andrew P. Morris, Lauren E. Petty, Craig L. Hanis, Nancy J. Cox, Adan Valladares-Salgado, Jennifer E. Below, Miguel Cruz

**Affiliations:** 1 Department of Anthropology, University of Toronto at Mississauga, Mississauga, ON, Canada; 2 Department of Genetics, Stanford University, Stanford, CA, United States of America; 3 School of Medicine, Stanford University, Stanford, CA, United States of America; 4 Department of Biostatistics, University of Liverpool, Liverpool, United Kingdom; 5 Department of Epidemiology, Human Genetics & Environmental Sciences, University of Texas School of Public Health, Houston, TX, United States of America; 6 Division of Genetic Medicine, Department of Medicine, Vanderbilt University, Nashville, TN, United States of America; 7 Unidad de Investigación Médica en Bioquímica, Hospital de Especialidades, Centro Médico Nacional Siglo XXI, IMSS, Mexico City, Mexico; Cincinnati Children's Hospital Medical Center, UNITED STATES

## Abstract

We carried out an admixture mapping study of lipid traits in two samples from Mexico City. Native American locus ancestry was significantly associated with triglyceride levels in a broad region of chromosome 11 overlapping the *BUD13*, *ZNF259* and *APOA5* genes. In our fine-mapping analysis of this region using dense genome-wide data, rs964184 is the only marker included in the 99% credible set of SNPs, providing strong support for rs964184 as the causal variant within this region. The frequency of the allele associated with increased triglyceride concentrations (rs964184-G) is between 30–40% higher in Native American populations from Mexico than in European populations. The evidence currently available for this variant indicates that it may be exerting its effect through three potential mechanisms: 1) modification of enhancer activity, 2) regulation of the expression of several genes in *cis* and/or *trans*, or 3) modification of the methylation patterns of the promoter of the *APOA5* gene.

## Introduction

Cardiovascular disease (CVD) is a leading cause of death worldwide, and is expected to remain a pressing health concern for at least the next two decades as current risk assessment fails to accurately capture the global lifetime burden of this disease [1,2]. Differences in CVD and stroke risk have been reported for various ethnic groups; Hispanic populations are among the most adversely affected by disease burden [3,4]. One of the major risk factors for CVD is dyslipidemia, estimated to affect 33% of the US population [5]. In particular, hypertriglyceridemia has been found to causally influence risk for CVD [6]. According to the National Health and Examination Survey (NHANES), Mexican Americans are characterized by higher prevalence of low HDL-C (high-density lipoprotein cholesterol), high LDL-C (low-density lipoprotein cholesterol), and high triglycerides when compared to White and Black US groups [7]. Rodriguez et al. [8]) alarmingly found that two-thirds of the Hispanic US population studied exhibited some form of dyslipidemia, with the highest incidence above 40 years of age. The high-triglyceride/low HDL-C dyslipidemia profile, which is described as atherogenic and linked to the metabolic syndrome, is particularly prevalent in this group [3,8–10]. In Mexico, the prevalence of dyslipidemias is also very high: a study based on the Mexican National Health and Nutrition Survey (ENSANUT 2006) indicated that the prevalence of total cholesterol concentrations ≥200 mg/dl was 43.6%, and the figures for LDL cholesterol ≥130 mg/dl, HDL cholesterol <40 mg/dl and triglycerides ≥150 mg/dl were 46%, 60,5% and 31.5%, respectively [11]. In Latin American populations, a strong association has been found between abnormal lipid profiles and increased risk of myocardial infarction [12]. With such a pronounced risk for dyslipidemia, it is critical to carry out more studies in Hispanic populations in order to understand the pathogenesis of this disease and its role in CVD.

Despite the need for ancestry-specific data, Hispanic populations have been underrepresented in lipid genomic studies [13–16] and clinical intervention trials [17], which have largely focused on European populations. Genome-wide association studies (GWAS) have great potential to elucidate the genetic architecture of dyslipidemia, but to date there have been only four GWAS of lipid traits in Hispanic populations [13,18–20]. Admixture mapping is an alternative approach to gene discovery in admixed populations, particularly when there is a differential distribution of disease traits or prevalence rates between the ancestral populations [21]. Admixture mapping tests for association of the relevant trait with locus ancestry, instead of testing for association with genotype, as is done in conventional GWA studies. The rationale behind the method is that, in chromosomal regions containing disease-risk variants, there is an expected overrepresentation of locus ancestry from the parental population with a higher proportion of risk alleles at the locus [22]. To our knowledge, research into the genetic basis of dyslipidemia utilizing this approach has not been completed in Hispanic populations. Here, we applied the admixture mapping method to identify genomic regions associated with lipid traits (total cholesterol, HDL-C, LDL-C, and triglycerides) in two samples from Mexico City. We also performed fine-mapping of the regions using dense genome-wide data available for the two Mexico City samples and a sample of Mexican Americans from Starr County, Texas.

## Results

Demographic characteristics, average lipid concentrations (HDL-C, LDL-C, TC, and TG) and average admixture proportions of the two samples, comprising 1310 and 1787 individuals from Mexico City, are displayed in Table 1.

**Table 1:** Descriptive information of the two Mexico City samples used for admixture mapping analysis. Values presented are averages with the standard deviation included in brackets. Average lipid concentrations do not reflect correction for lipid-lowering treatment.

	Mexico City 1	Mexico City 2
**Participants**	1310	1787
**Males:females**	477:833	892:895
**T2D cases:controls**	967:343	898:889
**Age**	50.57 (8.269)	52.78 (10.12)
**BMI**	29.06 (4.64)	28.64 (4.91)
**Total cholesterol (mg/dl)**	211.30 (43.38)	190.16 (44.73)
**HDL-C (mg/dl)**	46.04 (14.61)	45.19 (14.12)
**LDL-C (mg/dl)**	132.07 (35.15)	130.04 (34.59)
**Triglycerides (mg/dl)**	212.43 (142.11)	174.99 (113.39)
**Ancestry Proportions**		
**Native American**	0.6486	0.6233
**European**	0.3190	0.3525
**African**	0.0324	0.0242

The average Native American, European and African proportions estimated with the program RFMix were 64.86%, 31.90% and 3.24% for Sample 1, and 62.33%, 35.25% and 2.42% for Sample 2. A PCA plot depicting the two Mexican samples, in addition to relevant African, European, and Native American samples is shown in S1 Fig. Very strong correlations were observed when comparing individual admixture estimates obtained with the RFMix program and the ADMIXTURE program (Sample 1: R^2^ for NAM proportions = 0.9966, R^2^ for EUR proportions = 0.9953 and R^2^ for AFR proportions = 0.9322; Sample 2: R^2^ for NAM proportions = 0.9966, R^2^ for EUR proportions = 0.9950 and R^2^ for AFR proportions = 0.8537). We carried out admixture mapping using a linear regression model to evaluate the association of locus ancestry with lipid concentrations including average Native American ancestry, average African ancestry and diabetes status as covariates. Locus ancestry was defined as 0, 1, or 2 copies of Native American ancestry. Statistical significance was evaluated using a permutation-based approach that controls the familywise error rate (see Materials and Methods section for additional information). A region on chromosome 11 showed a significant association of Native American ancestry with TG concentrations in Sample 2 (beta = 0.164, p-value = 1.12x10^-6^, permutation p-value = 0.0060) and a borderline association in Sample 1 (beta = 0.169, p-value = 1.49x10^-5^, permutation p-value = 0.0660). The files depicting the full set of ancestry association results for each trait and sample are available in the Dryad Digital Repository (http://dx.doi.org/10.5061/dryad.7ns5c).

**Fig 1:**
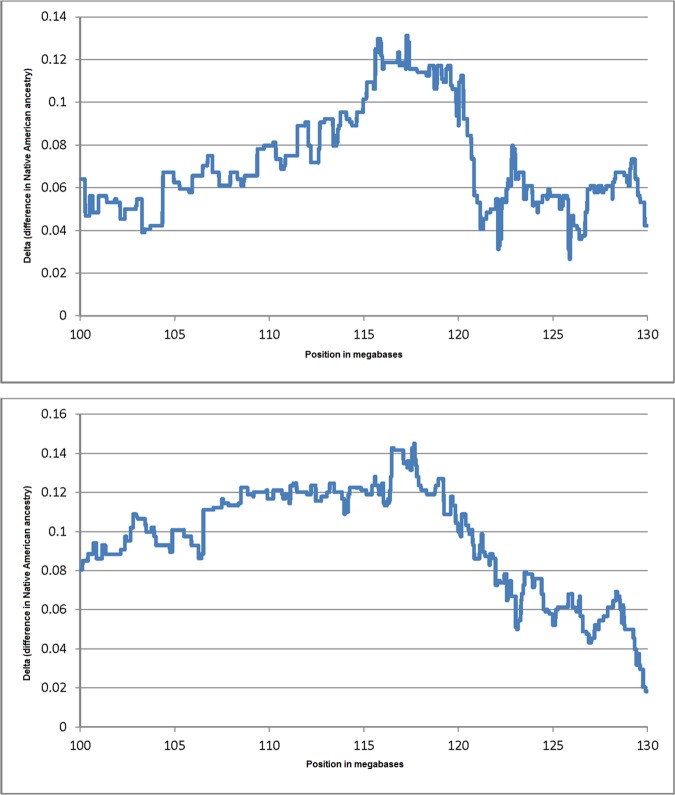
Plots showing the difference in Native American ancestry proportions between the individuals in the highest quartile of TG concentrations and individuals in the lowest quartile of TG concentrations. The plots show the differences observed for the region on chromosome 11 spanning from 100 Megabases to 130 Megabases. A/ Mexico City sample 1, B/ Mexico City sample 2.

The region identified in the admixture mapping scan is very large, encompassing several megabases, in which there is a positive association of Native American ancestry and TG concentrations. This region includes the genes *BUD13*, *ZNF259* and *APOA5*, and has been associated with TG concentrations in numerous GWA studies in different population groups (See Discussion section below). Based on these results, we carried out additional analyses focusing on this region. We divided both samples into quartiles based on TG concentrations, and compared the proportions of Native American ancestry between individuals in the highest and lowest quartiles. Fig 1 displays the differences in ancestry in this region for the two samples in the relevant genomic region. There are large differences in Native American ancestry between individuals in the highest and lowest quartiles of TG concentrations, with peak differences surpassing 12% and 14% NAM ancestry in Samples 1 and 2, respectively.

**Fig 2:**
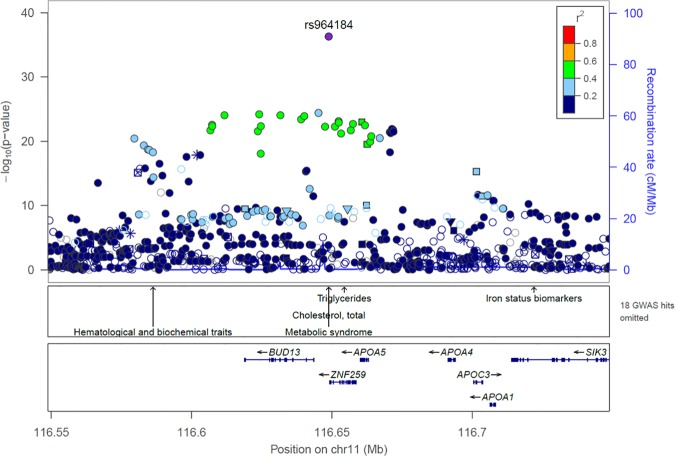
Regional plot showing the results of a meta-analysis of GWA studies in three Hispanic samples (Mexico City 1, Mexico City 2 and Starr County) for TG cholesterol. The region covers approximately 200 kb on chromosome 11, and includes the genes *BUD13/ZNF259/APOA5*.

In the next step, we carried out fine mapping based on a meta-analysis of GWA studies of lipid traits in three Hispanic samples: the two samples from Mexico City used for the admixture mapping study, and a sample from Starr County, TX. Detailed information about this meta-analysis can be found in Below et al. [20]. Importantly, this meta-analysis was based on data imputed with the very dense, sequence-based, Phase I 1000 Genomes reference samples and as expected, most of the common variants were imputed with good accuracy. On chromosome 11, less than 2.1% of the common markers (maf > 5%) had imputation scores < 0.5 in the Mexico City samples. **Fig 2** depicts the regional plot corresponding to the meta-analysis of GWA studies for TG in the region of chromosome 11 overlapping the *BUD13/ZNF259/APOA5* region. The lead SNP identified in this region was rs964184, with a p-value = 5.32x10^-37^. This is a G/C polymorphism and the G allele shows a strong association with TG concentrations. As can be seen in the plot, none of the other SNPs in the region have p-values < 1x10^-25^. Based on the results of the meta-analysis, we constructed a 99% credible set of SNPs (see Materials and Methods section for additional information). The only SNP in the 99% credible set is rs964184, indicating that this marker is driving the GWA signal observed in this region. If this is the case, we would expect that doing conditional analyses including this marker as a covariate would eliminate the signals observed in other SNPs within this region. This is indeed what we find when we do conditional analyses in both Mexico City samples. All the genome-wide significant signals that were observed in the original analyses in both samples disappear after conditioning for rs964184 (S1 Table).

Given that: 1) Native American ancestry shows a strong association with TG levels in this region, 2) the SNP rs964184 seems to be driving the signal observed in a meta-analysis of GWA studies for TG in this region, and 3) the allele G of rs964184 is strongly associated with increased TG concentrations, we would expect the G allele of rs964184 to have higher frequencies in Native American populations than in European populations. In European populations, the frequency of the G allele is between 12% and 20% (http://www.ncbi.nlm.nih.gov/projects/SNP/snp_ref.cgi?rs=964184; http://regulomedb.org/GWAS/rs964184_r2thr0.8_all.html). Unfortunately, there is very limited information about the allele frequencies of rs964184 in Native American populations. We estimated the frequency of the G allele in Native American populations using two alternative strategies: 1) extrapolating the allele frequency in the Native American parental population based on the frequencies observed in Europe (CEU sample), Africa (YRI sample), Mexico City, and the known admixture proportions of the Mexico City samples, which are known with good precision, and 2) genotyping the marker in Native American samples from Mexico (see Materials and Methods section for additional details). Using the first method, we estimated the Native American parental frequency to be approximately 50%, which is substantially higher than the European frequency (approximately 12% in the CEU sample). The genotyping of the marker in the indigenous samples from Mexico produced a remarkably concordant frequency for the G allele: 53.1% in the Nahua/Tlapanec sample from Guerrero, 53.5% in the Nahua sample from San Luis de Potosi, and 51.5% in the Teenek sample from Hidalgo.

The fine mapping analysis strongly pointed to rs964184 as driving the signal observed in this region, and as expected, we confirmed that the rs964184 allele associated with high TG concentrations has substantially higher frequencies in Native American than in European populations, thus potentially explaining the admixture mapping signal. Next, we carried out an annotation of this polymorphism to explore if there is any evidence pointing to potential regulatory effects. In RegulomeDB, this polymorphism has a score of 1f (eQTL + TF binding / DNase peak), indicating that this SNP may have a regulatory role. Annotation on Haploreg v4.1 also indicates that this polymorphism is an eQTL, alters fiver regulatory motifs, and overlaps with an enhancer in fat, liver and blood. Finally, the database rSNPBase also indicates that rs964184 may be involved in proximal transcriptional regulation.

## Discussion

### Admixture mapping identifies associations of locus ancestry with TG concentrations

Here, we present the results of an admixture mapping analysis of lipid traits in two independent samples from Mexico City. The two samples are characterized by substantial Native American and European ancestral contributions and very small African admixture (Table 1 and S1 Fig). We estimated locus ancestry using the program RFMix [23], using relevant reference samples from the parental populations. The Native American reference panels included samples from Mexico (Guerrero and Oaxaca), which is important in terms of obtaining proper estimates of Native American locus ancestry. The only region in which we observed genome-wide associations of locus ancestry with lipid traits was a broad region on chromosome 11, which showed a positive association of Native American ancestry with TG concentrations. This region includes the genes *BUD13/ZNF259/APOA5*, which have been associated with TG concentrations in multiple GWA studies and different population groups [13, 18–20, 24–34]. Ko et al. [13] also described an excess of Native American ancestry in this region for TG. These authors reported that individuals with high TG concentrations had approximately 5% higher Native American ancestry than individuals with low TG concentrations. When we compared the Native American admixture proportions in individuals in the highest quartile of TG concentrations and individuals in the lowest quartile of TG concentrations, we observed that individuals in the highest quartile had Native American proportions that were >10% higher than those in the lowest quartile (Fig 1).

### Fine-mapping points to rs964184 as the causal variant

The region showing significant excess of Native American ancestry in both Mexico City samples was very broad, spanning several megabases. In order to do fine mapping, we relied on the results of a meta-analysis of GWA studies focused on lipid traits that included the two samples from Mexico City used for this admixture mapping study, and a Mexican American sample from Starr County, TX [20]. The meta-analysis comprised 4,383 individuals. Importantly, the three GWA datasets used in the meta-analysis were imputed using the dense reference panels of the 1000 Genome Project Phase 1, so we were able to evaluate most of the common variants (e.g., those with minor allele frequencies > 1%) present in the three samples (the exception being the common variants with poor imputation scores, e.g. IMPUTE info scores < 0.5). The *BUD13/ZNF259/APOA5* region showed a genome-wide association signal for TG, and the lead SNP was rs964184 (p = 5.32x10^-37^) (Fig 2). Typically, due to the haplotype block structure of the human genome, regional plots of GWA studies show multiple markers with similar p-values to that of the lead SNP. However, in the regional plot of the *BUD13/ZNF259/APOA5* region for TG in our Hispanic samples, the p-value of rs964184 was substantially lower than the p-values of any other marker in the region. This is quite unusual, particularly considering that the dataset used for fine-mapping should be capturing most of the common variation present in this region. We explored in more detail the pattern of LD surrounding the SNP rs964184 using the LDlink webtool (http://analysistools.nci.nih.gov/LDlink/). This tool provides information about LD values (expressed as r^2^) between a query SNP and nearby variants, using information from Phase 3 of the 1000 Genomes Project. In the Mexican American sample from LA (MXL), there are no variants in strong LD (r^2^ > 0.8) with rs964184. The variant with the highest r^2^ value (0.7) is an indel (rs66505542) located in an intron of the gene *BUD13*, which was not present in our meta-analysis dataset. All the other variants have r^2^ values < 0.6 with rs964184. Therefore, the lack of variants in strong LD with rs964184 may explain the regional plot observed in our meta-analysis. Not unexpectedly given the large difference in p-values observed between rs964184 and all the other markers in the *BUD13/ZNF259/APOA5* region, the 99% credible set only includes one marker, rs964184, which is driving the GWA signal observed in this region.

### The marker rs964184 shows large frequency differences between European and Native American populations

Given the strong enrichment in Native American ancestry observed in the two Mexico City samples in the *BUD13/ZNF259/APOA5* region, the rs964184-G allele that is associated with high TG concentrations should be expected to have a higher allele frequency in Native American populations than in European populations. This is indeed the case. The allele frequency of rs964184-G in three Native American samples from Central Mexico (between 51.5% and 53.5%) is approximately 40% higher than the frequency observed in Europe (12% in the CEU sample). Therefore, the allele frequency data is consistent with the expectations of the admixture mapping results. Additionally, if rs964184 is responsible for the admixture mapping signal observed in both Mexican samples, we would expect that after adding rs964184 as a covariate to the statistical model in the admixture mapping analyses, the ancestry association would be dramatically attenuated or disappear. As expected, after conditioning for rs964184 the p-values for the first sample in the relevant region of chr11 go down from 1.45x10^-5^ (Empirical p-value 0.066) to 0.017 (Empirical p-value 1), and the p-values for the second sample go down from 1.12x10^-6^ (Empirical p-value 0.006) to 0.048 (Empirical p-value 1). These results are consistent with rs964184 driving the ancestry association signal, and the fine-mapping analysis (99% credible set) provides strong support for rs964184 being the causal marker in the *BUD13/ZNF259/APOA5* region.

### Association of rs964184 with TG concentrations in previous studies in Hispanic populations

It is important to note that in two previous GWA studies of lipid traits in Mexican samples [12,18] rs964184 was also the lead SNP for TG. In 2013, Weissglass-Volkov et al. [19] reported a strong association of rs964184 with TG concentrations in a sample from Mexico (5.5x10^-35^). Interestingly, the regional plot of the *BUD13/ZNF259/APOA5* region in this study was quite similar to that of our Mexican samples, with rs964184 having a substantially lower p-value that any other marker in the region. It should be noted that the imputations in this early study were based on the less dense HapMap reference panel, and consequently, the representation of common variants in the region was not complete. These authors then carried out cross-ethnic mapping using Mexican and European GWA datasets in order to refine the signals based on the differential patterns of LD in both groups, and suggested that rs964184 was the most plausible susceptibility variant in this region. They also investigated the effect of rs964184 on serum apoAV protein levels using an oral fat-tolerance test, and found that the rs964184-G allele was associated with postprandial response. During the response time (3–6 hours), the levels of apoAV protein, which are known to show an inverse relationship with TG levels, were significantly lower in individuals carrying the G allele. In Ko et al. [13], which similarly to our study, performed imputation using the dense 1000 Genomes Project samples, the lead SNP in the *BUD13/ZNF259/APOA5* region was also rs964184 (p = 6.08x10^-33^). They also reported the presence of an independent signal in the *SIK3* gene (lead SNP rs139961185, p = 1.15x10^-12^), which is in relatively low LD with rs964184 (r^2^<0.2). The authors carried out a detailed LD analysis in the region and identified a putative Mexican-specific TG risk haplotype, which tags two possible causal variants in this region, the non-synonymous variant rs3135506 and the variant rs662799, which is a strong enhancer in the HepG2 liver cell line. Given our dense dataset, we were able to evaluate these three variants in our Mexican samples. The SNP rs139961185 located in the *SIK3* gene was genome-wide significant in our samples (1.71x10^-8^). However, after conditioning on rs964184, the p-value of rs13996185 became non-significant (p = 0.922), indicating that, at least in our dataset, this variant is not independent of rs964184. Similarly, the p-values of rs3135506 and rs662799 were not significant after conditioning on rs964184. Therefore, in agreement with Weissglass-Volkov et al. [19], our interpretation is that rs964184 is the most plausible susceptibility variant in the *BUD13/ZNF259/APOA5* region.

### Potential mechanisms of action of rs964184

Annotation of rs964184 with several tools, such as RegulomeDB and Haploreg v4.1, indicate that this polymorphism may have a functional role. The variant is located in a DNaseI hypersensitive cluster, and it overlaps with an enhancer in several tissues, including liver and fat, which are relevant for lipid physiology. It is also possible that the effects of rs964184 are mediated through its role as an eQTL. In a recent study, Yao et al. [35] built a Cardiovascular Disease (CVD) network using more than 1,500 SNPs associated with 21 CVD traits, including TG. The authors then explored the role of these SNPs in whole blood gene expression using a large sample of 5,257 participants in the Framingham Heart Study. The authors classified rs964184 as a “hub SNP” associated with four different CVD-related traits: HDL-C, LDL-C, TG and CAD. This SNP was found to have *cis* associations with the genes *PCSK7*, *SIDT2*, *TAGLN*, and *BUD13* and *trans* associations with the genes *TMEM165*, *YPEL5*, *PPM1B* and *OBFC2A*. Using mediation analysis, the authors suggested that the genetic effect of rs964184 is mediated through its *cis* association with expression of *PCSK7* and *trans* association with expression of *PPM1B* and *YPEL5*. In particular, Yao et al. [35] found that expression of these three genes was not only associated with rs964184, but also with the concentrations of several lipid traits, including LDL-C and TG, in the Framingham Heart Study cohort, and suggested that these genes may represent potential therapeutic targets for lipid treatment. Another line of evidence pointing to rs964184 as the causal SNP in the *BUD13/ZNF259/APOA5* region is the report of genome-wide significant associations of this SNP with metabolites that share pathways with TG. Illig et al. [36] reported that rs964184 was associated with ratios of different phosphatidylcholines that are biochemically connected to TG by just a few enzymatic steps. These associations of rs964184 with phosphatidiylcholines have also been described in additional studies, such as Demirkan et al. [37], and Ganna et al. [38]. Finally, it is also important to note that rs964184 has also been associated with cytosine modification in two recent studies [39, 40]. Moen et al. [39] described rs964184 as an mQTL (modification quantitative trait loci) for a CpG in the promoter region of *APOA5*, which encodes apolipoprotein A-V). Pfeiffer et al. [40] also reported that rs964184 was associated with DNA methylation of a CpG in the promoter region of *APOA5*.

### Alternative interpretations

We note that there are other potential interpretations of these results, which we cannot eliminate given the characteristics of our dataset but are unlikely given the strong evidence reported above supporting rs964184 as the causal SNP. One interpretation would be that other common variants that were not present in our dataset or were poorly imputed may explain the signal observed in the *BUD13/ZNF259/APOA5* region. Our imputations were done using the 1000 Genomes Project Phase 1 reference panels, and as expected most of the common variants were imputed with good accuracy in the Mexico City samples. Additionally, none of the poorly imputed common markers (info scores < 0.5) had p-values lower than that observed for rs964184. However, as described above, the indel rs66505542, which is the only variant with r^2^ values > 0.7 with rs964184 in the Mexican American LA (MXL) 1000 Genomes Project sample was not present in the Mexico City datasets.

Another interpretation would be that low-frequency variants (0.5%-5%) or rare variants (maf<0.5%) may be driving the signal observed in the *BUD13/ZNF259/APOA5* region. The sample size of the meta-analysis used for fine mapping was lower than 4,500 individuals, so this dataset was not powered to identify low-frequency or rare variants. We believe that it is unlikely that the signal observed in the *BUD13/ZNF259/APOA5* region is driven exclusively by low-frequency (0.5%-5%) and more particularly rare variants (maf<0.5%). It would be difficult to explain the large excess of Native American ancestry in the region (>10% higher in individuals in the highest quartile of TG concentrations with respect to individuals in the lowest quartile) as a result of the effect of rare variants: this would require the presence of multiple rare variants of Native American ancestry increasing TG concentrations. We performed formal rare variant tests in a subset of the Starr County cohort with whole-exome sequence data to determine if rare variants could explain the signal at rs964184 in this sample, and no significant association with *ZNF259* or *APOA5* was observed, lending further support that rare variants do not drive this signal. Also, it is important to note that two very large studies in European populations [28,34] have identified rs964184 as the lead SNP in the *BUD13/ZNF259/APOA5* region, the most recent one based on dense imputed datasets [34]. Rare variants tend to be structured geographically, and it is unlikely that rs964184 would be tagging rare variants present in both European and Mexican populations. In the most recent meta-analysis in European populations, Surakka et al. [34] implemented gene-based tests to identify association of lipid traits with accumulations of minor alleles at well-imputed rare variants. Two genes in the *BUD13/ZNF259/APOA5* region, *ZNF259* (also known as ZPR1) and *APOA5*, were significantly associated with TG concentrations. Conditional analyses adjusting for rs964184 reduced the strength of the association of rare variants in both genes (in *ZNF259*, from 1.4x10^-11^ to 3.3x10^-4^; in APOA5, from 4.7x10^-8^ to 4.3x10^-5^), although could not fully explain the effect of the mutational load. We downloaded the summary data of Surakka et al. [34] for the *BUD13/ZNF259/APOA5* region (available at http://diagram-consortium.org/2015_ENGAGE_1KG/). The regional plot of this region in Europeans is remarkably similar to that in the Mexican samples (Fig 2). The p-value for rs964184 is substantially lower than the p-values observed for any other marker in the region (p = 1.75x10^-157^ for rs964184 vs. 8.80x10^-107^ for the next most significant marker, rs3741298). Similarly to the results observed in our samples, in the European dataset the 99% credible set only includes rs964184, strongly suggesting that rs964184 is the causal variant in this region.

It has also been described in the literature that in some cases, due to the mutational history of a particular region, the lead SNP may be capturing the effect of more than one functional common SNP. Because of this, the lead SNP may have substantially lower p-values than the functional SNPs, even if it is not the causal variant. A good example of this are the signals observed in the *CYP2C9* region in GWA studies focused on warfarin dosage. There are two well-known common non-synonymous variants within the *CYP2C9* gene (CYP2C9*2 and CYP2C9*3) that have a strong effect on the required dosage of warfarin, the most commonly used anticoagulant worldwide. However, in GWA studies in European [41] and Brazilian populations [42], the lead SNPs in this region are markers that capture the joint effects of both polymorphisms. For example, in the recent Brazilian study [42], the top signal was the SNP rs9332238 (p = 4.4x10^-13^), whereas the p-values of CYP2C9*2 and CYP2C9*3 did not reach genome-wide significance (p = 2.1x10^-7^ and p = 1.8x10^-5^, respectively). A detailed analysis of LD between the three markers showed that the minor allele of rs9332238 is always associated with CYP2C9*2 or CYP2C9*3 (but never with both), and this explains that rs9332238 has a lower p-value than the two functional SNPs. It is not possible to know if this example could be extrapolated to the *BUD13/ZNF259/APOA5* region, because the functional variants in this region remain to be discovered. Overall, based on the evidence available for rs964184 (fine mapping and 99% credible set in Hispanic and European samples, allele frequency differentiation between European and Native American groups, annotation data, association with prostprandial response in an oral fat-tolerance test in a Mexican sample, and lack of evidence indicating that rare variants may be driving the TG signal) in our view the most plausible explanation is that rs964184 is the causal SNP in the *BUD13/ZNF259/APOA5* region.

### Study limitations

Finally, it is important to note some of the limitations our study. The sample size in the discovery (admixture mapping) stage was relatively small, so our study only has statistical power to identify regions with large effects. Additionally, the GWA datasets used in the fine mapping stage of our project included the two Mexico City samples employed in the discovery (admixture mapping) stage. This is something that is conventionally done in admixture mapping studies but it may be a source of bias. However, we would like to emphasize that independent studies in other samples from Mexico and in other population groups have also identified rs964184 as the lead signal within the *BUD13/ZNF259/APOA5* region.

### Conclusions

In this study we identified a significant association of Native American ancestry with TG concentrations in a broad region of chromosome 11 overlapping the *BUD13/ZNF259/APOA5* genes. Fine-mapping analyses strongly point to rs964184 as the causal variant. The frequency of rs964184-G, the allele that has been associated with increased TG concentrations in multiple population groups, is substantially higher in Native American populations than in European populations, and this may explain the admixture mapping signal observed in this region. This polymorphism may be exerting its effect through three potential mechanisms: 1) modification of enhancer activity, 2) regulation of the expression of several genes in *cis* and/or *trans*, or 3) modification of the methylation patterns of the promoter of the *APOA5* gene. Further evidence is needed to clarify these issues.

## Materials and methods

### Study participants

The admixture mapping study was carried out in two independent samples from Mexico City. Sample 1 is composed of 967 individuals with type 2 diabetes (664 females and 303 males) and 343 controls (169 females and 174 males). Sample 2 is composed of 898 individuals with type 2 diabetes (536 females and 362 males) and 889 controls (359 females and 530 males). The samples were collected for a GWAS of type 2 diabetes. More detailed information about the samples is provided in Parra *et al*. [43] and Below *et al*. [20]. Written Informed consent was obtained from each participant and the research was approved by the ethical research board of the Medical Center ‘Siglo XXI’.

### Measurement of plasma lipid concentrations

Quantification of total cholesterol, HDL-C, LDL-C, and triglycerides was carried out with an IIab 300 Plus clinical chemistry analyser (Instrument Laboratory, Lexington, MA 02421–3125, USA), using standard protocols. The serum samples were obtained after fasting for at least 12 hours and the biochemical quantification took place within 3 hours of serum purification.

### Genotyping

Sample 1 was genotyped with the Affymetrix Genome-wide Human SNP array 5.0 (Affymetrix, Santa Clara, CA) following standard protocols. Genotype calling was done with the Affymetrix PowerTools (APT) software package including the full sample set, and using two genotyping algorithms, the BRLMM-P and Birdseed algorithms. In order to minimize genotyping errors, the program PLINK v 1.06 was used to merge the genotype results obtained with both algorithms, using the consensus call mode.

Sample 2 was genotyped with the Affymetrix Axiom LAT array (Affymetrix, Santa Clara, CA) following standard protocols. Genotype calling was done with the Affymetrix PowerTools (APT) software package, using the AxiomGT1/ BRLMM-P algorithm and the manufacturer recommended calling pipeline.

### Quality control

Prior to admixture mapping, SNPs were removed from the initial list of autosomal markers based on set criteria. For Sample 1, the criteria were: 1) minor allele frequency <1%, 2) Hardy-Weinberg p-values <0.0001 in the control group, 3) missingness >5% in the cases and the controls and 4) A/T or C/G polymorphisms. The final number of markers used for admixture mapping was 302,860. For Sample 2, the criteria were: 1) Markers classified as *CallRateBelowThreshold*, *OffTargetVariants* or *Other* by the program SNPolisher, 2) minor allele frequency <1%, 3) Hardy-Weinberg p-values <0.01 in controls and 4) A/T or C/G polymorphisms. The final numbers of markers used for admixture mapping was 329,163.

### Inference of local ancestry

We ran the discriminative ancestry classifier RFMix [23] to estimate local ancestry for each position on the genome. As the two strata of the data used different arrays, we estimated ancestry separately for each dataset using similar protocols. We modeled the admixture including ancestry from Europeans, Africans and Native Americans, to accurately model the relevant components of ancestry typically seen across Mexican populations [44,45]. The CEU and YRI populations from 1000 Genomes Phase 1 were chosen for European and African ancestry, respectively, across both arrays. For the Affymetrix 5.0 data, we included a set of Nahua, Maya, Quechua and Aymara as Native American representatives. We subset these to the 42 individuals with minimal European ancestry previously used for local ancestry estimation in the 1000 Genomes Project. For the Affymetrix LAT array samples, we included a set of 95 Native Mexican samples from Oaxaca, which primarily include individuals of Mixe, Mixtec and Zapotec ancestry [46]. These individuals were used to seed the “Pop-phased” (switch error-corrected) version of RFMix, followed by 5 rounds of iteration to refine local ancestry estimates. For all other parameters in the algorithm we used the default calls. After local ancestry estimation, we reformatted the ancestry estimates into diploid local ancestry calls for downstream analyses.

### Transformation of lipid phenotypes prior to admixture mapping

Prior to the statistical analyses, plasma lipid concentrations were adjusted based on the effect of drug-lowering medications, as suggested by Wu et al. [47]. In the two Mexico City samples analyzed here, the effect sizes and p-values of the main signals observed in the GWAS study are quite similar using the full sample with adjustment for drug-lowering medications, or only the individuals without treatment (data not shown). After adjustment of lipid values based on medication, we applied a rank-based inverse normal transformation to obtain normally distributed values for the subsequent admixture mapping scan. Briefly, we ran a linear regression model with lipid concentrations (total cholesterol, HDL-C, LDL-C, and triglycerides) as dependent variables, and sex, age, age2, and BMI as independent variables. The residuals of this regression were inverse normal transformed with the Blom method. These transformed residuals were used as dependent variables in the admixture mapping analysis, as described below.

### Admixture mapping

Admixture mapping association analysis was conducted in PLINK using a linear regression model with transformed lipid concentrations as dependent variables and locus ancestry (defined as 0, 1 or 2 copies of Native American ancestry), global Native American and African ancestry and diabetes status as independent variables. Given that the current Mexican population is the result of a recent admixture process, the chromosomal ancestral segments identified by the program RFMix are quite large, and there is a high correlation of ancestry estimates between markers. For this reason, using a Bonferroni correction based on the total number of markers is not appropriate in this situation. We tested statistical significance using an approach based on label-swapping permutation testing using the Max(T) approach, which controls the family-wise error rate. The empirical p-values obtained were based on 10000 permutations.

### Fine mapping

For the significant regions identified in both samples using the admixture mapping approach, we carried out fine mapping using dense imputed data available for the two Mexico City samples and a Mexican American sample from Starr County, Texas. These three samples of Mexican ancestry were recently used to carry out a GWA study for lipid traits [20], and comprise 4,383 individuals. The samples were imputed using the combined 1000 Genomes Phase 1 integrated variant set as the reference panel. Briefly, in the three samples, a linear regression was run using the lipid concentrations as dependent variables and sex, age, age2, BMI, relevant PC scores and diabetes status as covariates. The residuals were then inverse normal transformed using the Blom method. The transformed residuals were tested with the program SNPTEST v2 based on an additive model, using the *score* method to take into account genotype uncertainty. The program META was used to carry out an inverse variance fixed-effects meta-analysis. Detailed information is available in Below et al. [20].

Based on the effect sizes and standard deviations observed for the markers present in the region of interest, a 99% credible set of SNPs (e.g. the set of SNPs that are likely to be driving the GWAs signal based on the statistical evidence of association) was constructed using the approach developed by Wakefield [48]. Briefly, we calculated the posterior probability that the j^th^ variant, π_Cj_, is driving a distinct association signal by

$$\pi_{Cj}=\frac{{Ʌ}_{j}}{{Ʃ}_{k}{Ʌ}_{k}}$$

where the summation is over all variants in the fine-mapping region. In this expression, Λ_j_ is the approximate Bayes’ factor for the j^th^ variant, given by

$${Ʌ}_{j}=\left[\sqrt{\frac{V_{j}}{V_{j}+\omega }}\right]\;\exp\;\left[\frac{\omega \beta_{j}^{2}}{2V_{j}\;(Vj+\omega )}\right]$$

where β_j_ and V_j_ denote the allelic effect and corresponding variance from the meta-analysis for the association signal. The parameter ω denotes the prior variance in allelic effects, taken here to be 0.04. A 99% credible set was then constructed by: (i) ranking all variants according to their Bayes’ factor, Λ_j_; and (ii) including ranked variants until their cumulative posterior probability exceeds 0.99.

### Annotation

The variants identified during fine mapping were annotated using a variety of tools, including Haploreg, RegulomeDB and rSNPBase.

### Principal components analysis

The program EIGENSOFT [49] was used to perform a principal components analysis, after pruning markers showing short-range and long-range linkage disequilibrium using a threshold of r^2^ = 0.2.

### Estimating rs964184 allele frequencies in Mexican indigenous populations

In the fine mapping effort, we identified rs964184 as the lead SNP in the GWAs for TG in the *BUD13/ZNF259/APOA5* region (see Results section below). Due to the limited information about rs964184 in American indigenous populations, we estimated the allele frequencies of this polymorphism in two independent samples of indigenous groups from Mexico. The first sample comprises 32 unrelated individuals of Nahua and Tlapanec ancestry from the state of Guerrero. Written informed consent was obtained from the participants and the sample collection was approved by the Internal Review Board of Penn State University. The second sample includes 46 unrelated individuals of Nahua ancestry from the state of Hidalgo and 97 unrelated individuals of Teenek ancestry from the state of San Luis de Potosi. These individuals also provided written informed consent and the protocol approved by the Institutional Review Board of the National Ethical Committee of the Instituto Mexicano del Seguro Social (IMSS).

### Analysis of rare variants based on exome-sequence data from Starr County

To test for rare variant association with the genes of interest, association tests were performed using GRANVIL in a subset of 1,460 samples for whom whole-exome sequence data was available in the Starr County cohort. For each lipids phenotype, association tests were run with and without controlling for imputed rs964184 dosages and both i) including all variants with minor allele frequency less than 0.01 in *ZNF259* and *APOA5* and ii) including only the subset of variants predicted to be functional.

## Supporting information

S1 Fig Principal Component Analysis (PCA) of the Mexican samples used in the admixture mapping analysis and relevant parental populations samples. (TIF)

S1 Table P-values obtained in TG GWA study for markers in the *BUD13/ZNF259/APOA5* region with and without conditioning for rs964184. (XLSX)
