# Supplementary figures and images for: Admixture mapping in two Mexican samples identifies significant associations of locus ancestry with triglyceride levels in the BUD13/ZNF259/APOA5 region and fine mapping points to rs964184 as the main driver of the association signal
Source: PLoS One. 2017 Feb 28;12(2):e0172880. doi: 10.1371/journal.pone.0172880 (PMC5330487; doi:10.1371/journal.pone.0172880)

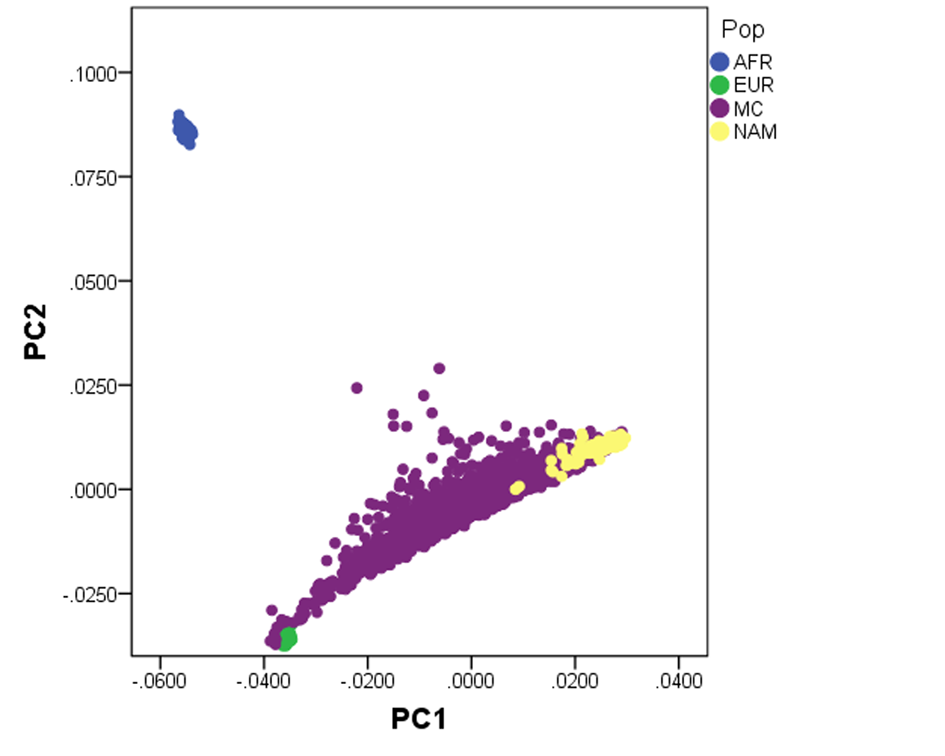

Supplement: S1 Fig — (TIF) [file pone.0172880.s001.tif]
